# Real-world implementation of lecanemab and donanemab in an Italian memory center: a 1-year experience

**DOI:** 10.1186/s13195-026-02015-6

**Published:** 2026-03-20

**Authors:** Federica Agosta, Giordano Cecchetti, Edoardo G. Spinelli, Alma Ghirelli, Giulia Rugarli, Stefano Pisano, Federico Coraglia, Elisa Canu, Veronica Castelnovo, Elisa Sibilla, Anna Gilioli, Chiara Tripodi, Fabiola Freri, Alessandra Bianchi, Paolo Vezzulli, Sonia Calloni, Andrea Falini, Ana Maria Samanes Gajate, Andrea Panzacchi, Gino Pepe, Camilla Ferri, Arturo Chiti, Massimo Filippi

**Affiliations:** 1 Center for Alzheimer’s and Related Diseases (CARD), Neurology Unit, IRCCS San Raffaele Scientific Institute, Milan, Italy; 2 Neuroimaging Research Unit, Division of Neuroscience, IRCCS San Raffaele Scientific Institute, Via Olgettina, 60, Milan, 20132 Italy; 3 Vita-Salute San Raffaele University, Milan, Italy; 4 Neurotech Hub, Vita-Salute San Raffaele University, Milan, Italy; 5 Neuroradiology Service, IRCCS San Raffaele Scientific Institute, Milan, Italy; 6 Nuclear Medicine Service, IRCCS San Raffaele Scientific Institute, Milan, Italy; 7 Pharmacy Unit, IRCCS San Raffaele Scientific Institute, Milan, Italy; 8 Neurorehabilitation Unit, IRCCS San Raffaele Scientific Institute, Milan, Italy

**Keywords:** Alzheimer’s disease, Lecanemab, Donanemab, Real-world evidence, Plasma biomarkers, Amyloid-related imaging abnormalities (ARIA)

## Abstract

**Background:**

Anti-amyloid monoclonal antibodies are entering clinical practice for early symptomatic Alzheimer’s disease (AD), but European real-world data on feasibility, safety, and biomarker monitoring remain limited. We report the first year of implementation of lecanemab and donanemab in an Italian tertiary memory center.

**Methods:**

We conducted a prospective observational real-world cohort study at the Center for Alzheimer’s and Related Diseases of IRCCS Ospedale San Raffaele (Milan, Italy). Twenty-nine treatment courses were administered in patients with early symptomatic AD (lecanemab, *n* = 9; donanemab, *n* = 20) under European Medicines Agency–aligned safety monitoring and risk-mitigation protocols. Given the unbalanced treatment groups, results are descriptive and not intended for direct comparison between treatments. Safety surveillance included serial magnetic resonance imaging for amyloid-related imaging abnormalities (ARIA) and systematic recording of infusion-related reactions. Biological monitoring included amyloid positron emission tomography with Centiloid quantification and plasma biomarkers (phosphorylated tau 181 and 217, glial fibrillary acidic protein, neurofilament light chain, and amyloid-β 42/40 ratio) at baseline and follow-up. Baseline comparisons and longitudinal changes were assessed using appropriate parametric or nonparametric statistical methods.

**Results:**

ARIA were infrequent. In donanemab-treated patients, mildly symptomatic ARIA-E occurred in 10% (2/20) and asymptomatic ARIA-H in 15% (3/20). In lecanemab-treated patients, asymptomatic ARIA-H occurred in 11% (1/9). Infusion-related reactions occurred in 21% (6/29) of treatment courses and were manageable with standardized premedication. Among 11 patients with six-month follow-up, amyloid burden decreased significantly (mean change − 52.4 Centiloids), and 75% of donanemab-treated patients (6/8) and 0% of lecanemab-treated patients reached amyloid positron emission tomography negativity (< 11CL). Plasma phosphorylated tau 181 and glial fibrillary acidic protein showed directional declines, consistent with expected biomarker trajectories under anti-amyloid therapy, while cognitive measures showed no significant change.

**Conclusions:**

In a structured multidisciplinary framework, lecanemab and donanemab were feasibly implemented with a preliminary favorable early safety profile, substantial amyloid reduction, and measurable plasma biomarker changes in routine practice. This experience supports the feasibility of structured real-world pathways for deployment and monitoring of disease-modifying therapies in European memory clinics, within the limitations of a small cohort and short follow-up.

## Background

Anti-amyloid monoclonal antibodies have recently transformed the therapeutic landscape for early symptomatic Alzheimer’s disease (AD), defined in clinical and regulatory frameworks as mild cognitive impairment (MCI) or mild dementia due to AD with confirmed amyloid pathology [1, 2]. Lecanemab and donanemab were shown to substantially reduce amyloid burden and to produce statistically significant, albeit modest, slowing of cognitive and functional decline under controlled phase 3 trial conditions, leading to European regulatory review processes and positive CHMP opinions for use in early symptomatic AD with confirmed amyloid pathology [3, 4]. Their introduction into routine care, however, requires the establishment of specialized infrastructures capable of managing eligibility assessment, infusion logistics, and safety surveillance, particularly regarding amyloid-related imaging abnormalities (ARIA).

Real-world evidence (RWE) is crucial to characterize how these therapies can be operationalized outside controlled trial settings. In this context, the most relevant domains include implementation feasibility, safety governance (particularly ARIA surveillance and management), and biological target engagement under routine clinical conditions, rather than comparative efficacy assessment. Initial RWE from the United States and Israel has shown that treatment implementation is logistically complex and ARIA rates vary across settings, underscoring the need for structured multidisciplinary governance [5–7]. Yet, European data remain limited, and reports integrating MRI monitoring, quantitative amyloid-PET, plasma biomarkers, and cognitive trajectories within a coordinated clinical framework are scarce.

In 2024, the Center for Alzheimer’s and Related Diseases (CARD) of IRCCS Ospedale San Raffaele (Milan, Italy) established a comprehensive anti-amyloid program aligned with EMA appropriate-use recommendations (AUR). The program integrates multidisciplinary expertise and structured safety surveillance, including standardized MRI monitoring, quantitative Centiloid (CL) PET, and longitudinal plasma biomarker assessment.

The present study reports one of the first European real-world experience with both lecanemab and donanemab delivered through a multidisciplinary model. The objectives were to: (i) assess feasibility and safety, including the frequency and management of ARIA and infusion-related reactions; (ii) quantify early amyloid removal using CL PET; (iii) characterize plasma biomarker trajectories; and (iv) evaluate short-term cognitive outcomes. These data provide insights into the translational performance of anti-amyloid monoclonal antibodies in routine practice and may inform structured approaches to the implementation of disease-modifying therapies for AD within European care networks.

## Methods

### Study design and setting

This prospective, observational real-world cohort study was conducted at the CARD of IRCCS Ospedale San Raffaele (Milan, Italy), a tertiary referral memory clinic with a structured multidisciplinary program for the administration of amyloid targeting therapies (ATTs). The cohort was embedded within a pre-structured clinical program in which safety monitoring (serial MRI), biological assessments (baseline and 6-month amyloid-PET and plasma biomarkers), and cognitive evaluations were predefined components of routine care. Data were prospectively recorded within the clinical pathway and subsequently analyzed for the present study. Between September 2024 and October 2025, patients received lecanemab or donanemab under EMA-aligned protocols and Italian regulatory provisions for named-patient access. All infusions were performed in short-stay hospital wards under neurologist supervision, with standardized procedures for pre-infusion evaluation, infusion monitoring, and post-infusion observation. The treatment program integrates neurology, neuroradiology, nuclear medicine, neuropsychology, pharmacy, and administrative services, enabling a unified diagnostic–therapeutic workflow from referral to longitudinal monitoring [8]. The center also implemented institutional educational sessions for radiology, emergency, and neurology staff focusing on ARIA detection and acute management.

### Participants and eligibility assessment

Eligible participants met clinical criteria for MCI or mild dementia due to AD, corresponding to stages 3 and 4 of the 2024 NIA-AA clinical staging [9–11]. Biological confirmation of amyloid pathology was required through cerebrospinal fluid (CSF) biomarkers or amyloid-PET. For CSF confirmation, AD pathology was defined according to locally validated Lumipulse cut-offs: amyloid-β 42/40 ratio (Aβ42/40) < 0.069; phosphorylated tau 181 (pTau181) > 56.5 ng/L; and total tau (tTau) > 506 ng/L [9]. Amyloid-PET positivity at screening was determined by standardized visual read. Quantitative CL values were systematically derived and used both for baseline characterization and for longitudinal treatment monitoring, including protocol-based discontinuation criteria for donanemab [1, 12]. Only ApoE ε4 non-carriers or heterozygotes were eligible, in accordance with EMA restrictions at the time of treatment initiation [3, 4]. This regulatory constraint influenced cohort composition and limits generalizability to homozygous ε4 carriers. Exclusion criteria included: >4 cerebral microbleeds or any superficial siderosis on baseline GRE MRI sequences; history of stroke or TIA within the previous 12 months; any prior seizure; moderate-to-severe small-vessel disease (Fazekas > 2); anticoagulant therapy; suspected CAA-related inflammation; or significant medical or psychiatric instability interfering with adherence and safety [10, 11].

Patient selection followed a structured multi-step workflow. Initial screening, conducted in outpatient clinics or via telemedicine, included review of clinical history, prior biomarker documentation, and preliminary assessment of cognitive–functional status [8]. When telemedicine was used, structured interviews with patients and caregivers and review of prior neuropsychological reports were performed. When biological confirmation was not yet available, AD CSF biomarkers were used as first-line diagnostic tools unless lumbar puncture was contraindicated. Subsequent evaluations included a comprehensive standardized neuropsychological battery administered according to the institutional protocol, ApoE genotyping, and a 1.5T MRI performed within six months using 3DT1-weighted, 3DFLAIR, T2-weighted, GRE, SWI and DWI sequences. The same neuropsychological battery was repeated at follow-up to ensure longitudinal comparability across cognitive domains.

All patients underwent baseline amyloid-PET for quantitative CL assessment, with tracer consistency maintained throughout follow-up. PET images were processed using validated tracer-specific calibration models within the cNeuro^®^ cPET platform (Combinostics Oy, Tampere, Finland) [13].

Final treatment eligibility was determined through shared decision-making among the treating neurologists. Complex or ambiguous cases were reviewed in multidisciplinary meetings with neuroradiologists, nuclear medicine specialists, and neuropsychologists. Before therapy initiation, patient and caregiver understanding, willingness, and ability to comply with infusion schedules and MRI surveillance were systematically verified.

### Treatment procedures

Lecanemab was administered at 10 mg/kg intravenously every two weeks [3, 4]. Donanemab was administered every four weeks following a titration scheme of 350 mg, 700 mg, 1050 mg, and 1400 mg, in line with EMA-approved protocols [1, 4, 11, 14].

Infusions were conducted in dedicated hospital areas with medical oversight and standardized emergency equipment.

Following early infusion-related reactions (IRRs) in initial patients, a uniform premedication protocol was implemented for both antibodies [6]: methylprednisolone 125 mg IV, cetirizine 10 mg PO, famotidine 40 mg PO, and acetaminophen 1 g PO before the first four infusions, with tapering individualized thereafter. Infusion rates were reduced or temporarily paused in case of IRRs.

### Safety monitoring and ARIA management

Serial MRI monitoring followed EMA product information [3, 4]: for lecanemab, scans were performed at baseline and before the 5th, 7th, and 14th infusions; for donanemab at baseline and before the 2nd, 3rd, 4th, and 7th doses. Additional MRI was obtained for any new neurological symptoms. MRI was acquired on 1.5T scanners (3DFLAIR, T2-weighted, GRE, SWI and DWI sequences), consistent with routine clinical practice. The combined use of GRE and SWI sequences aimed to optimize detection of microhemorrhages and superficial siderosis [8]. ARIA-E and ARIA-H were classified by trained neuroradiologists using standardized criteria [15].

ARIA management followed AUR [10, 11]. Mild asymptomatic ARIA-H permitted therapy continuation with closer monitoring. Moderate ARIA-E or symptomatic mild ARIA led to temporary suspension, with monthly MRI until resolution. Severe ARIA, or ARIA with severe symptoms, required definitive discontinuation. Symptomatic cases were treated with high-dose intravenous corticosteroids, following institutional safety protocols.

As part of the safety governance framework, a 24/7 direct contact line was available for acute symptom reporting, and all patients received a Medical Alert Card indicating ongoing anti-amyloid therapy, ARIA risk, and contraindications to anticoagulants and thrombolytics. For patients residing outside Milan, pre-identified local neurologists and MRI facilities ensured rapid triage.

IRRs were prospectively recorded at each infusion and graded according to severity criteria outlined in the AUR [10, 11], distinguishing mild (grade 1), moderate (grade 2), and severe (grade ≥ 3) reactions based on symptom intensity, need for infusion interruption, and pharmacological intervention. Management followed these predefined severity-based algorithms.

### Longitudinal clinical and biological monitoring

In addition to MRI surveillance for ARIA detection, all patients participated in a structured follow-up program for longitudinal clinical and biological assessment.

Amyloid-PET was performed at baseline and at 6 months. For donanemab-treated patients, PET negativity was operationally defined as < 11 CL, in accordance with the protocol-based discontinuation criterion adopted in our clinical program [1]. In addition, a < 24 CL threshold, commonly used in clinical trials to define amyloid clearance, was applied descriptively in secondary analyses to facilitate comparison with trial-based definitions [1, 2].

At baseline and 6 months, blood samples were collected for quantification of pTau181, phosphorylated tau 217 (pTau217), glial fibrillary acidic protein (GFAP), neurofilament light chain (NfL), and the Aβ42/40 ratio using the Lumipulse G600II automated platform [16]. Samples were processed within the institutional biobank (the Biological Resource Center: CRB-OSR; BBMRI-ERIC ID: bbmri-eric: ID: IT_1383758011993577) following standardized preanalytical procedures to minimize variability.

Neuropsychological assessment was performed using a standardized comprehensive battery administered identically at baseline and 6 months. Core measures included MMSE, CDR, and CDR-SB. Additional domains systematically assessed comprised memory (e.g., RAVLT, Rey complex figure recall, short story recall), executive and attentional functions (e.g., digit span, Trail Making Test A–B, verbal fluency, Stroop), language (e.g., Token Test, naming and comprehension tasks), visuospatial abilities (e.g., Rey figure copy, Clock Drawing Test), and praxis. Caregiver-based instruments included ADL/IADL scales, Neuropsychiatric Inventory, extended CDR, and quality-of-life measures. The full list of instruments and corresponding references is provided in Additional File 1.

Given the progressive implementation and staggered enrollment within the treatment program, follow-up duration varied across participants; six-month data were available only for individuals who had reached this time point at the October 2025 data lock.

### Statistical analysis

Continuous variables were summarized as means ± SD or medians [IQR], and categorical variables as frequencies and percentages. Between-group comparisons used Welch’s t-test or Fisher’s exact test. Within-subject longitudinal changes were analyzed using paired t-tests or Wilcoxon signed-rank tests. Standardized response means (SRMs) were calculated to quantify effect sizes. Given the exploratory real-world design and limited sample size, all analyses were considered descriptive and hypothesis-generating rather than confirmatory. Moreover, no formal comparative analyses between lecanemab and donanemab were performed. Statistical significance was set at *p* < 0.05 (two-sided). Analyses were conducted in R v4.4.1.

## Results

### Patient inclusion and baseline characteristics

During the observation period, all consecutive patients referred to the CARD of IRCCS Ospedale San Raffaele for ATT evaluation were prospectively screened within the structured referral pathway. Although the exact denominator of screened individuals could not be reconstructed, a substantial proportion was excluded at initial clinical assessment, most commonly due to advanced disease stage or caregiver overestimation of functional autonomy. Financial considerations also represented a relevant barrier, as treatment costs were entirely patient-borne.

Among patients who completed the full diagnostic work-up for ATT eligibility (*n* = 44), 28 ultimately initiated therapy (63.6%), accounting for 29 treatment courses overall, as one patient transitioned from lecanemab to donanemab after limited amyloid-PET response at 6 months. Six additional patients (13.6%) were considered eligible but declined treatment due to financial constraints. Ten patients (22.7%) were classified as screening failures after completion of the diagnostic work-up. Reasons for post–work-up exclusion included: absence of biological amyloid pathology (*n* = 2), despite a clinically typical amnestic presentation; clinically significant co-pathology (*n* = 1 with Lewy body disease features supported by positive DAT-scan); incidental MRI findings (*n* = 1 glioma); excessive microbleed burden on screening MRI (*n* = 2; >4 microbleeds on GRE); and advanced disease stage exceeding recommended treatment thresholds (*n* = 4; CDR > 1 or equivalent functional impairment). In discordant cases between GRE and SWI sequences, GRE was prioritized for eligibility assessment following multidisciplinary discussion, in accordance with current AUR [10, 11, 15].

According to the 2024 NIA-AA staging, 14 individuals within the treated cohort (50%) were classified as stage 3 (including 6 early-onset AD [EOAD], 4 late-onset AD [LOAD], 1 logopenic variant of primary progressive aphasia [PPA], 1 mixed PPA, 2 posterior cortical atrophy [PCA]) and 14 (50%) as stage 4 (5 EOAD, 8 LOAD, 1 PCA). At initiation, 9 patients received lecanemab (7 ongoing at data lock) and 20 received donanemab (14 ongoing). The mean waiting time between first neurological evaluation and treatment start was 12 ± 3 weeks.

**Fig. 1:**
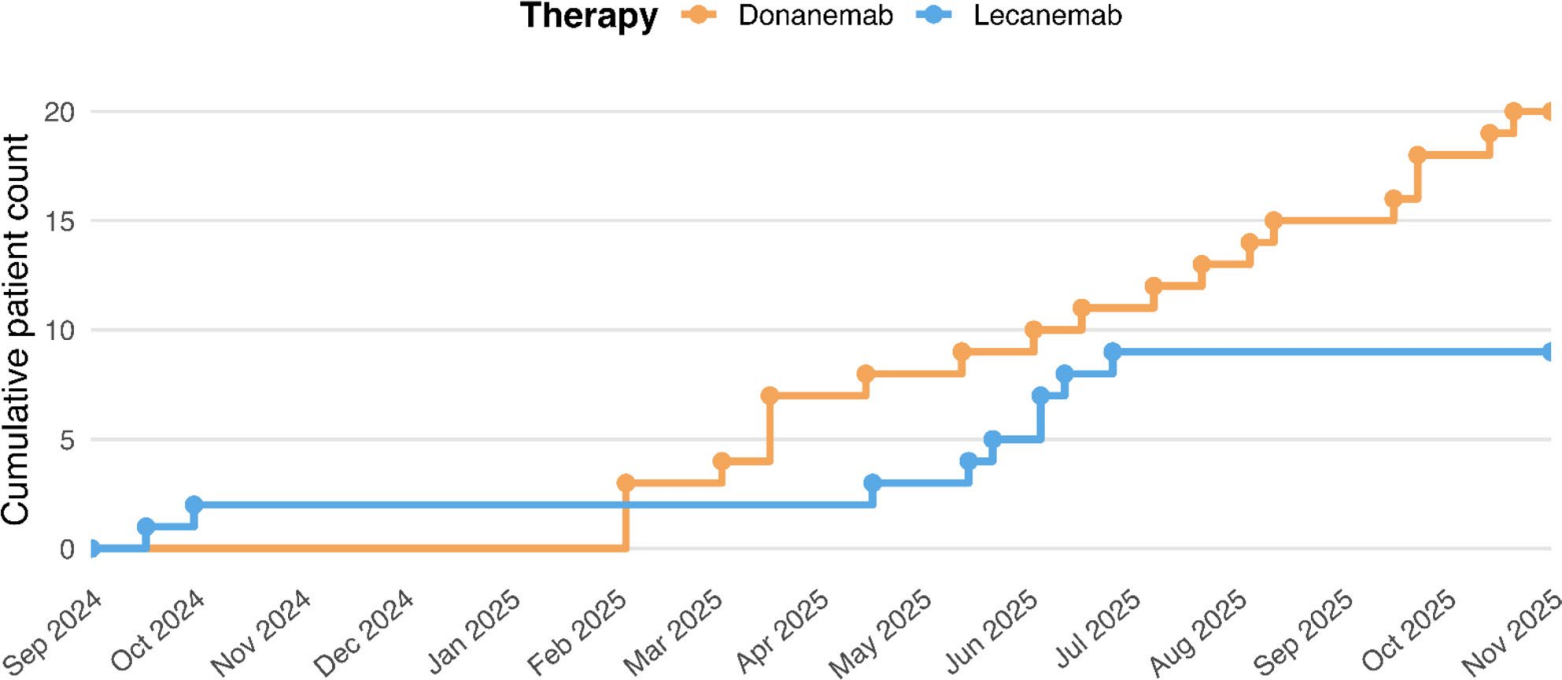
Cumulative initiation of anti-amyloid therapy by drug. Cumulative curves display the progressive number of treatment courses (*n* = 29) initiated with lecanemab (*n* = 9, blue) or donanemab (*n* = 20, orange) from the start of the disease-modifying therapy program through October 2025. The figure illustrates the temporal adoption of each therapy across the cohort

Baseline characteristics are reported in Table 1. All patients had biologically confirmed amyloid pathology, with a baseline CL value of 93.1 ± 22.4, consistent with moderate-to-high plaque burden. Lecanemab recipients were more frequently ApoE ε4 carriers, reflecting early program phases when all heterozygotes were assigned to lecanemab prior to CHMP positive opinion for donanemab. Baseline cognitive performance was comparable between groups. MMSE values ranged from 16 to 29; six patients had scores < 20. In these cases, CDR global ratings (0.5–1.0) and CDR-SB profiles were consistent with early symptomatic AD. Importantly, MMSE was not used as a standalone exclusion criterion; eligibility was determined through an integrated cognitive–functional assessment aligned with 2024 NIA-AA staging rather than predefined MMSE cutoffs. Apart from ApoE distribution, no other significant group differences were observed (all *p* > 0.05). Figure 1 illustrates cumulative ATT initiations.

**Table 1:** Baseline demographic, clinical, and biomarker characteristics of the treated cohort

Variable	All (*n* = 29)	Lecanemab (*n* = 9)	Donanemab (*n* = 20)	*p*-value
Age, years	67.4 ± 7.8 (55.9–82.8)	67.8 ± 8 (56.4–79.2)	67.2 ± 7.8 (55.9–82.8)	0.849
Female, %	55%	67%	50%	0.454
APOE ε4 carriers, %	34%	78%	15%	**0.002**
Education, years	16.3 ± 4.8 (5–27)	15.9 ± 2.3 (12–18)	16.5 ± 5.6 (5–27)	0.680
Disease duration, months	40.7 ± 18.8 (13–88)	42.6 ± 12.9 (17.6–60.2)	39.9 ± 21.2 (13–88)	0.722
Vascular risk factors, n	0.9 ± 0.9 (0–3)	1.1 ± 1.1 (0–3)	0.8 ± 0.9 (0–3)	0.527
Hypertension, %	45%	33%	50%	0.454
Cognitive features at baseline				
MMSE, score	24.1 ± 4.1 (16–29)	22.8 ± 4.8 (16–29)	24.7 ± 3.8 (17–29)	0.305
CDR, global	0.7 ± 0.2 (0.5–1.0)	0.7 ± 0.2 (0.5–1.0)	0.7 ± 0.3 (0.5–1.0)	0.745
CDR-SB, score	3.4 ± 1.7 (1.0–7.0)	3.5 ± 1.9 (1.0–6.5)	3.4 ± 1.6 (1.0–7.0)	0.869
Neuroimaging and fluid biomarkers at baseline				
Amyloid-PET Centiloid (baseline)	93.1 ± 22.4 (45.1–135)	94.1 ± 16.7 (59.5–118.7)	92.6 ± 25 (45.1–135)	0.857
Microhemorrhages (mHs)	0.6 ± 0.6 (0–2)	0.4 ± 0.5 (0–1)	0.6 ± 0.6 (0–2)	0.490
Plasma GFAP (baseline, pg/mL)	104.7 ± 50.1 (6.6–282.5)	109.3 ± 29.6 (44.4–143.5)	102.4 ± 58.4 (6.6–282.5)	0.689
Plasma NfL (baseline, pg/mL)	26.5 ± 9.9 (14–52.6)	30.9 ± 12.4 (18.2–52.6)	24.3 ± 7.9 (14–46.1)	0.167
Plasma pTau-181 (baseline, pg/mL)	2.3 ± 1.1 (0.3–4.6)	2.4 ± 0.9 (1.1–3.3)	2.3 ± 1.2 (0.3–4.6)	0.803
Plasma pTau-217 (baseline, pg/mL)	0.872 ± 0.54 (0.203–1.993)	0.918 ± 0.578 (0.263–1.731)	0.849 ± 0.536 (0.203–1.993)	0.767
Plasma Aβ42/40 (baseline)	0.07 ± 0.01 (0.033–0.087)	0.07 ± 0.006 (0.062–0.080)	0.069 ± 0.012 (0.033–0.087)	0.847
CSF pTau-181 (baseline, ng/mL)	127.7 ± 74.7 (47–298.3) (*n* = 19)	149 ± 74.6 (65–268.2) (*n* = 7)	115.2 ± 75.1 (47–298.3) (*n* = 12)	0.360
CSF total Tau (baseline, ng/mL)	790.4 ± 428.1 (276–1834) (*n* = 19)	932.6 ± 404.7 (506–1726) (*n* = 7)	707.5 ± 436.1 (276–1834) (*n* = 12)	0.276
CSF Aβ42/40 (baseline)	0.043 ± 0.01 (0.028–0.06) (*n* = 19)	0.04 ± 0.012 (0.028–0.059) (*n* = 7)	0.045 ± 0.009 (0.031–0.06) (*n* = 13)	0.355

Continuous variables are presented as mean ± standard deviation (SD) and range (min–max); categorical variables as percentages. p-values reflect between-group comparisons (Welch’s t-test for continuous variables; Fisher’s exact or χ² test for categorical variables). CSF biomarkers were available only in a subset of patients; the number of available cases is reported in parentheses in the table. CSF cut-offs for amyloid positivity: Aβ42/40 < 0.069; pTau181 > 56.5 ng/L; total tau > 506 ng/L. Amyloid-PET positivity determined by visual read. Quantitative Centiloid values were systematically derived and used both for baseline characterization and for longitudinal treatment monitoring, including protocol-based discontinuation criteria for donanemab

*Abbreviations*: *Aβ* Amyloid-β, *ApoE* Apolipoprotein E, *CDR* Clinical Dementia Rating, *CDR-SB* Clinical Dementia Rating–Sum of Boxes, *GFAP* Glial fibrillary acidic protein, *MMSE* Mini-Mental State Examination, *NfL* Neurofilament light chain, *pTau* Phosphorylated tau

### Treatment exposure

**Fig. 2:**
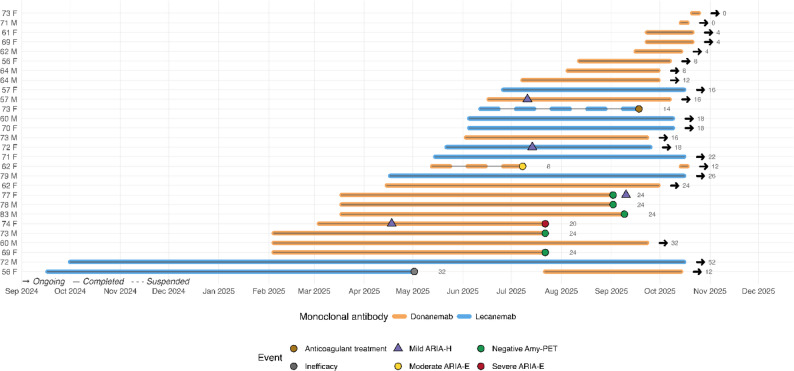
Individual treatment timelines for patients receiving anti-amyloid therapy. Each horizontal bar represents a single treatment course. Blue and orange segments denote periods on lecanemab and donanemab, respectively. Continuous lines indicate ongoing therapy, and dashed lines indicate suspended courses. Clinical events are marked by colored circles: green = amyloid-PET negativity (therapy discontinued per protocol); red = severe ARIA-E; yellow = moderate ARIA-E; brown = temporary interruption due to anticoagulant therapy; grey = discontinuation for inefficacy. Right-pointing arrows indicate ongoing treatment at data lock (October 2025). One patient who showed minimal amyloid reduction on lecanemab is represented by a transition from blue to orange, reflecting the switch to donanemab. Patients are ordered by treatment initiation date, with age and sex reported alongside each row

Across the cohort, patients received a median of 5 (range 1–9) donanemab infusions over 14 (0–32) weeks and 10 (8–27) lecanemab infusions over 18 (14–52) weeks. Eleven patients completed six months of therapy with repeat amyloid-PET, plasma biomarkers, and cognitive testing (Fig. 2). One patient completed twelve months of therapy.

### Safety

#### Amyloid-related Imaging Abnormalities (ARIA)

Among 20 donanemab-treated patients, ARIA-E occurred in 2 cases (10%), both symptomatic (one moderate and one severe, the latter associated with ARIA-H). ARIA-H were observed in 3/20 patients (15%), predominantly asymptomatic; two cases were isolated mild ARIA-H and one occurred in association with severe ARIA-E (see Table 2).

**Table 2:** Safety events and treatment outcomes during anti-amyloid therapy

Event	Donanemab (*n* = 20)	Lecanemab (*n* = 9)
ARIA-E (ever), n (%)	2 (10%)	0
ARIA-H (ever), n (%)	3 (15%)	1 (11%)
• ARIA-H without ARIA-E (ever)	2	1
• ARIA-H with ARIA-E (ever)	1	0
Infusion-related reactions, n (%)	3 (15%)	3 (33%)
Serious adverse events, n (%)	2 (10%)	0
Treatment suspension, n (%)	1 (5%) – moderate ARIA-E (until radiological resolution)	1 (11%) – anticoagulant therapy after fracture
Treatment discontinuation, n (%)	6 (30%) – 5 for PET negativity at month 6; 1 severe ARIA-E/H	1 (11%) – limited amyloid clearance
Deaths	0	0

Summary of safety events and treatment outcomes in patients treated with donanemab and lecanemab. Percentages refer to number of patients. “Ever” indicates occurrence at any time during follow-up

*Abbreviations*: *ARIA*  Amyloid-related imaging abnormality, *ARIA-E*  ARIA with edema/effusion, *ARIA-H*  ARIA with hemosiderin deposition, *IRR*  Infusion-related reaction, *PET*  Positron emission tomography

A 74-year-old woman (mixed PPA, APOE ε3/ε3, baseline CL 48) developed a severe ARIA-E/H after the sixth infusion, following an asymptomatic mild ARIA-H at the second dose. MRI demonstrated extensive multifocal ARIA-E with numerous new microbleeds and new superficial siderosis foci. She was hospitalized, treated with high-dose corticosteroids, and permanently discontinued donanemab. Neurological findings (left homonymous hemianopia) resolved, and follow-up MRI at three months showed near-complete ARIA-E resolution with stabilization of ARIA-H.

A 61-year-old woman (EOAD, APOE ε3/ε3, baseline CL 109) developed moderate multifocal ARIA-E after the third infusion, detected after a fall with dizziness. She was hospitalized for monitoring, treated with corticosteroids, and resumed donanemab after complete radiological resolution at two months using a slower titration schedule.

Two additional donanemab patients developed isolated mild ARIA-H and remained asymptomatic, continuing treatment without interruption.

Among nine lecanemab-treated patients, one mild, isolated, asymptomatic ARIA-H (11%) was detected; no ARIA-E occurred.

Two additional donanemab-treated patients underwent emergency evaluation for transient neurological symptoms; MRI and EEG were unremarkable, and symptoms resolved spontaneously.

#### Infusion-related Reactions (IRRs) 

IRRs occurred mainly during the first two infusions in 6 of 29 treatment courses (21%), involving 33% of lecanemab-treated patients and 15% of donanemab-treated patients (Table 2) [10, 11].

Lecanemab IRRs typically manifested as Grade 2 fever/chills, according to the predefined AUR severity grading [10, 11]; one patient experienced recurrent Grade 3 fever requiring targeted allergology consultation and short-course oral steroids, with complete resolution in subsequent infusions. Donanemab IRRs presented as fever, chills, myalgias, flushing, or transient hypotension; all resolved with supportive therapy, corticosteroids, and/or infusion-rate adjustments. No Grade ≥ 4 reactions or hospitalizations due to IRRs occurred.

### Longitudinal clinical and biological monitoring

Given the clinical heterogeneity of the cohort (including EOAD, LOAD, and atypical phenotypes such as PPA and PCA) and the limited sample size, longitudinal biological and cognitive outcomes are presented as pooled descriptive analyses and subgroup findings should be considered descriptive.

#### Amyloid-pet findings 

Among the 11 patients who completed six-month amyloid-PET follow-up, amyloid burden decreased significantly across the cohort (mean − 52.4 CL; 95% CI − 68.8 to − 36.0; SRM − 1.89; *p* < 0.001)(Table 3).

**Table 3:** Cognitive and amyloid-PET changes over 6 months of anti-amyloid therapy (*n* = 11)

Measure	Baseline	6 Months	Mean Δ (95% CI)	SRM	*p*-value
MMSE	24.6 ± 4.1	24.2 ± 4.1	-0.45 [− 1.8, + 0.9]	-0.2	0.52
CDR	0.7 ± 0.3	0.6 ± 0.2	-0.05	-0.1	0.48
CDR-SB	3.0 ± 1.5	3.3 ± 1.6	+ 0.28 [− 0.1, + 0.5]	0.44	0.18
Amyloid-PET (Centiloids)	89.9 ± 28.2	22.4 ± 33.9	-52,4 [-68.8, -36.0]	-1.89	**< 0.001**
Amyloid-PET (Centiloids), by age group					
< 65 years (*n* = 3)	104.8 ± 13.6	68.0 ± 30.1	-36.8 [-64.1, -9.5]	-1.52	0.12
> 65 years (*n* = 8)	63.5 ± 35.6	5.2 ± 12.4	-58.3 [-77.7, -38.8]	-2.08	**< 0.001**

Values are expressed as mean ± SD or mean change (95% confidence interval). Standardized response means (SRM) quantify within-subject effect sizes. Paired t-tests were used to assess longitudinal changes. Exploratory subgroup analysis by age (< 65 vs. ≥ 65 years) revealed numerically greater amyloid reduction in older participants, although between-group comparisons were not performed given the small sample size and short-term follow-up

*Abbreviations*: *MMSE*  Mini-Mental State Examination, *CDR*  Clinical Dementia Rating, *CDR-SB*  CDR Sum of Boxes, *PET*  Positron emission tomography, *SRM*  Standardized response mean

**Fig. 3:**
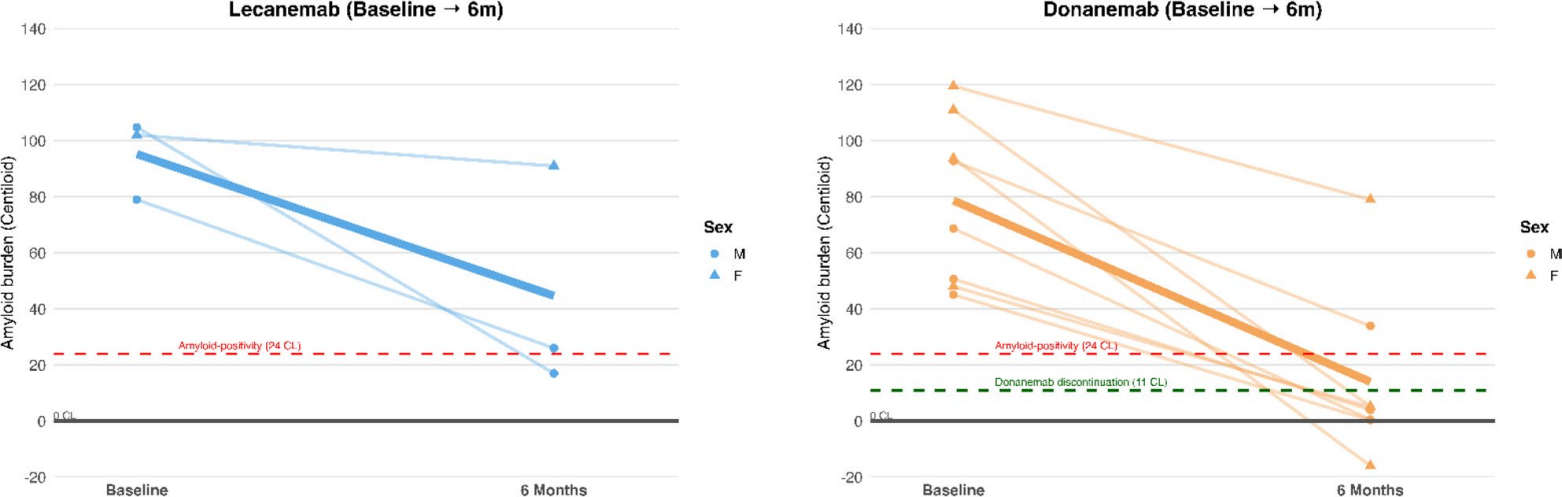
Longitudinal amyloid-PET trajectories by treatment group. Spaghetti plots depict individual changes in cortical amyloid burden (Centiloid units) from baseline to follow-up among patients treated with donanemab (orange) and lecanemab (blue). Thin lines represent individual trajectories, and thick lines represent median values. Circles and triangles denote male and female participants, respectively. Dashed horizontal lines indicate thresholds for amyloid positivity (24 CL, red) and donanemab discontinuation (< 11 CL, dark green). Donanemab recipients showed robust amyloid reduction, with several achieving PET negativity at 6 months, whereas lecanemab-treated individuals demonstrated more heterogeneous responses, including one case with minimal reduction who subsequently switched to donanemab

Among donanemab-treated patients (*n* = 8), baseline CL averaged 93 ± 25, with a mean reduction of − 64.7 ± 28.1 at six months (Fig. 3). Six individuals (75%) achieved PET negativity (< 11 CL), meeting discontinuation criteria.

Among lecanemab-treated patients (*n* = 3), baseline CL was 94 ± 17, with a mean reduction of − 50.6 ± 38.5. Two showed marked amyloid removal (e.g., 104.8→17 CL), while one patient demonstrated minimal response (102→91 CL) and switched to donanemab after completion of five lecanemab half-lives [11].

Given subgroup imbalance, no formal between-group comparisons were performed. Exploratory age-stratification suggested greater amyloid clearance in patients ≥ 65 years (− 58 ± 20 CL) compared with < 65 years (− 37 ± 28 CL), as reported in Table 3.

#### Plasma biomarkers

**Fig. 4:**
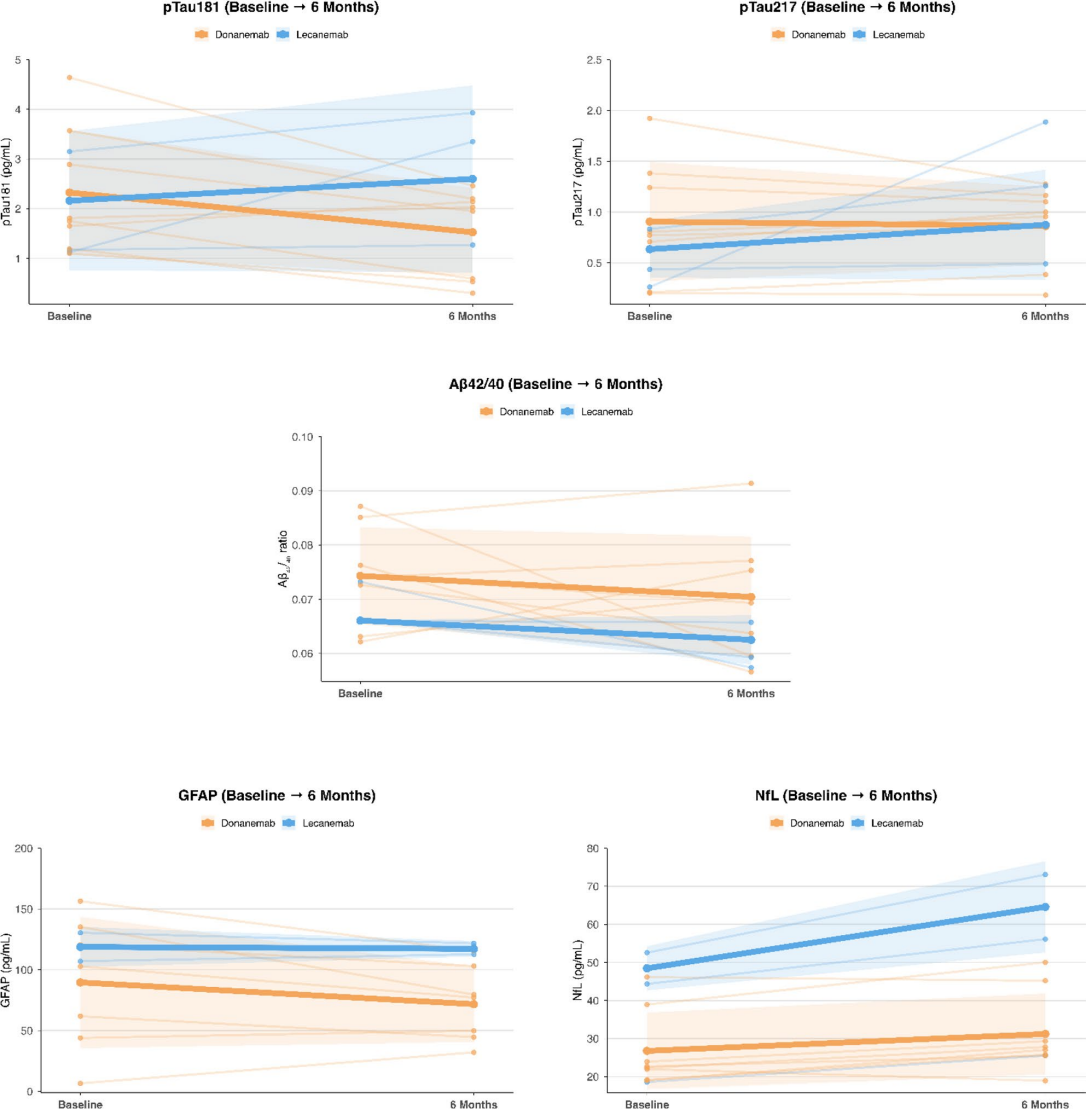
Plasma biomarker changes over 6 months by treatment group. Spaghetti plots show individual trajectories (thin lines) and group mean ± SD (thick lines and shaded ribbons) for plasma GFAP, NfL, pTau181, pTau217, and Aβ₄₂/₄₀ ratio from baseline to 6 months. The lecanemab non-responder (who subsequently switched to donanemab) was excluded from mean ± SD estimates to avoid distortion of group-level trends. These plots illustrate the longitudinal biological response to treatment across key plasma biomarkers

Donanemab-treated patients (*n* = 8) showed a numerical decrease in GFAP (− 20 ± 26 pg/mL; *p* = 0.066), consistent with a trend-level change and a significant reduction in pTau181 (− 0.8 ± 0.9 pg/mL; *p* = 0.033), together with a mild increase in NfL (+ 4.4 ± 4.7 pg/mL; *p* = 0.031) (Fig. 4). pTau217 and Aβ42/40 remained overall stable.

Among lecanemab-treated patients (*n* = 2 ongoing), changes were modest; however, the patient with 12-month follow-up demonstrated robust amelioration across all biomarkers. The non-responder switching from lecanemab to donanemab showed worsening biomarker profile at month 6.

#### Cognitive outcomes

Across 11 patients with 6-month follow-up, cognition remained stable (Table 3). MMSE showed no significant change (mean Δ − 0.45; *p* = 0.52). CDR global scores were unchanged, and CDR-SB exhibited minimal non-significant variation (mean Δ + 0.28; *p* = 0.18).

One lecanemab-treated patient with 12-month follow-up maintained stability (MMSE 29→28; CDR 0.5; CDR-SB + 0.5).

## Discussion

This prospective real-world analysis provides one of the first European experiences with the structured implementation of ATTs for early AD in routine clinical practice. Conducted within a tertiary referral memory center equipped with multidisciplinary governance and EMA-aligned safety infrastructure, this study suggests that donanemab and lecanemab can be safely implemented in routine practice with measurable biological target engagement. Given the exploratory design and limited sample size, and unbalanced treatment groups, these findings are descriptive and are not intended to support direct quantitative comparisons with randomized clinical trials or between donanemab and lecanemab within this cohort.

Across 29 treatment courses, ARIA incidence remained low and manageable. In donanemab-treated patients, ARIA-E occurred in 10% and ARIA-H in 15%, including three mild asymptomatic ARIA-H and two moderate-to-severe ARIA-E requiring treatment interruption and corticosteroid therapy. Both cases improved with high-dose corticosteroids and showed radiologic resolution within 8–12 weeks. Lecanemab demonstrated excellent tolerability, with only one mild isolated ARIA-H (11%) and no ARIA-E. No macrohaemorrhages, recurrent ARIA, or treatment-related deaths occurred. All monitoring MRIs were performed on 1.5T systems; although consistent with routine clinical practice, the use of 3T MRI may offer greater sensitivity for microhemorrhage detection in research settings.

The favorable ARIA profile likely reflects both pharmacologic factors and center-level governance. Donanemab’s stepwise titration (350→700→1050→1400 mg) has been shown to reduce ARIA-E risk by ~ 40% without compromising amyloid clearance [14, 17]. Lecanemab tolerability aligned with CLARITY-AD and with real-world reports from Israel and the United States, which described ARIA frequencies around 10–20%, predominantly mild and asymptomatic [2, 5–7]. However, these comparisons should be interpreted cautiously, as ARIA frequencies may be influenced by sample size, differential ApoE ε4 selection, regulatory eligibility constraints, and structured monitoring intensity. Our cohort was restricted to ε4 non-carriers or heterozygotes and reflects real-world regulatory conditions, which may differ from pivotal trial populations and other real-world settings.

Clinical governance also played a crucial role. Standardized MRI surveillance aligned with EMA product information, monthly multidisciplinary ARIA-review meetings, and a 24/7 neurology contact line enabled rapid safety triage. A regional referral network ensured emergency MRI access for out-of-region patients [3, 4]. Each patient received a Medical Alert Card detailing contraindications to thrombolytics and anticoagulants, an essential precaution given emerging thrombolysis-related haemorrhage reports in ATT recipients.

Two patients with acute neurological symptoms underwent prompt evaluation (with emergency MRI/EEG), were ARIA-negative, and recovered spontaneously. While anecdotal, these events illustrate the operational importance of rapid triage pathways and imaging availability in real-world treatment programs. The overall framework (integrating neurology, neuroradiology, nuclear medicine, and neuropsychology) may provide a potentially scalable model for safe regional expansion of ATTs.

IRRs occurred in 21% of treatment courses (33% lecanemab; 15% donanemab), were mostly mild-to-moderate, and responded promptly to supportive therapy and corticosteroids. Early implementation of a standardized premedication protocol effectively reduced recurrence. This IRR frequency fits between pivotal-trial rates and recent United-States real-world data, where ~ 30–37% of lecanemab-treated patients experienced IRRs before premedication strategies were incorporated [1, 2, 5, 6]. Israeli real-world evidence similarly identified IRRs as common early events responsive to local premedication protocols [7]. Collectively, our findings confirm that IRRs are typically early, manageable, and rarely severe in clinical practice.

Biological target engagement was substantial and occurred early during treatment. After six months, cortical amyloid burden decreased by − 52.4 CL (95% CI − 68.8 to − 36.0; SRM − 1.89; *p* < 0.001). This early and marked clearance likely reflects early treatment initiation, optimized scheduling, and consistent ARIA surveillance that prevented delays.

In the subset of patients with six-month PET follow-up, donanemab-treated individuals showed marked amyloid reduction (mean − 65 CL), with 75% meeting predefined PET negativity criteria (< 11CL). Lecanemab-treated patients demonstrated a mean − 51 CL reduction, with two near-complete responders and one minimal responder who subsequently switched to donanemab after pharmacologic washout. Given the limited sample size, no formal analysis of clinical or genetic predictors of rapid amyloid clearance was performed. Descriptive inspection did not reveal a consistent pattern across sex, APOE ε4 status, or ARIA occurrence.

Amyloid-PET served not only a diagnostic but also a treatment-monitoring role. In our experience, a six-month PET appeared useful for stratifying response trajectories: (i) rapid clearance enabling discontinuation (donanemab), (ii) near-complete clearance predicting negativity at 12 months, and (iii) inadequate clearance suggesting suboptimal response. For lecanemab, where therapy continuation is independent of PET, interim PET may help identify patients with limited biological response. The safe switch from lecanemab to donanemab illustrates the potential feasibility of PET-informed treatment adjustment within this cohort, although this strategy requires validation in larger prospective dataset.

Plasma biomarkers demonstrated directionally consistent changes. In donanemab recipients, GFAP (− 20 ± 26 pg/mL; *p* = 0.066) and pTau181 (− 0.8 ± 0.9 pg/mL; *p* = 0.033) declined, while NfL modestly increased (+ 4.4 ± 4.7 pg/mL; *p* = 0.031). This pattern may be consistent with early treatment-related disease-modifying effects and with neuroaxonal dynamics, although the mechanistic interpretation remains speculative within the limits of our dataset. pTau217 and Aβ42/40 remained stable. These patterns are directionally consistent with biomarker changes reported in randomized trials [1, 2], suggesting that GFAP and pTau181 are sensitive early markers of target engagement, underscoring their potential value for longitudinal monitoring in clinical settings. Among lecanemab-treated patients, trajectories were heterogeneous: two had minimal change, whereas one patient with 12-month follow-up demonstrated broad biomarker normalization. The non-responder transitioning to donanemab showed marked pTau elevation at six months, consistent with delayed or absent amyloid-target engagement.

Cognitive outcomes remained stable over six months in the 11 patients with available follow-up. MMSE, CDR, and CDR-SB showed no significant deterioration (all *p* > 0.05), with small effect sizes (SRM − 0.2 to 0.4). Given the limited sample size and short observation period, these findings should be interpreted as preliminary and descriptive rather than indicative of clinical efficacy. This stability aligns with expectations for early symptomatic patients over short intervals. In pivotal phase 3 trials [3, 4], cognitive separation between active treatment and placebo was already observable within the first months, although formal inferential analyses were prespecified for longer endpoints (i.e., 18 months). Therefore, the stability observed in our cohort over six months is directionally consistent with early trial trajectories, while acknowledging differences in design and sample size. One lecanemab-treated patient with 12-month data showed sustained cognitive stability (MMSE 29→28; CDR 0.5; CDR-SB + 0.5), paralleling imaging and biomarker improvements. Longer observation will clarify whether early biomarker and imaging changes translate into lasting cognitive and functional benefit in real-world practice.

Age-related variability emerged as an exploratory signal. In our cohort, participants aged ≥ 65 years showed numerically greater amyloid reduction than younger individuals, despite higher baseline amyloid burden in the latter group [18]. In pivotal donanemab analyses, greater baseline amyloid burden has been associated with larger absolute amyloid reductions and differential probability of achieving PET negativity [19, 20]. However, our age-stratified comparison was exploratory, based on a small and unbalanced sample, and not adjusted for baseline CL values or exposure parameters. The shorter follow-up duration compared with phase 3 trials may also influence apparent subgroup differences. Therefore, the observed age-related pattern should be interpreted cautiously and does not imply a true age-dependent biological effect. Larger real-world datasets with formal modeling of baseline burden and exposure will be required to clarify potential interactions between age and amyloid clearance dynamics.

Notably, no patient was lost to follow-up. All individuals either completed therapy or discontinued per protocol (after ARIA or PET negativity). This full retention underscores both patient adherence and the operational feasibility of ATTs within a well-structured academic network.

The main limitations are sample size and limited follow-up duration, which constrain predictive modeling and long-term cognitive conclusions. In addition, during the study period ATTs were not reimbursed within the Italian National Health System and were entirely patient-funded. Therefore, the proportion of eligible patients initiating therapy may not reflect uptake rates under publicly reimbursed conditions and should not be generalized to healthcare systems with established reimbursement pathways. Nonetheless, the cohort reflects the heterogeneity of real-world memory-clinic populations and offers insights into early implementation of disease-modifying therapies. Ongoing extensions will assess longer-term imaging–plasma coupling, durability of amyloid suppression, and clinical outcomes post-discontinuation.

## Conclusions

This study suggests that ATTs can be feasibly and safely delivered within a structured multidisciplinary framework in European routine practice, within the limitations of a single-center cohort. The convergence of safety, imaging, plasma, and cognitive findings supports the feasibility of translating disease-modifying therapies into appropriately structured real-world care pathways. Beyond this single-center experience, the rollout of ATTs will require structured reorganization of dementia networks, as emphasized in recent European and Italian consensus frameworks [21–23].

## Data Availability

Data sharing is restricted due to patient privacy, regulatory requirements, and institutional policies. Data access may be considered on a case-by-case basis by the corresponding author.

## Abbreviations

AD: Alzheimer’s disease
Aβ: Amyloid-β
ApoE: Apolipoprotein E
ARIA: Amyloid-related imaging abnormalities
ARIA-E: Amyloid-related imaging abnormalities with edema/effusion
ARIA-H: Amyloid-related imaging abnormalities with hemorrhage
ATT: Amyloid targeting therapy
AUR: Appropriate Use Recommendations
CAA: Cerebral amyloid angiopathy
CDR: Clinical Dementia Rating
CDR-SB: Clinical Dementia Rating–Sum of Boxes
CL: Centiloid
CSF: Cerebrospinal fluid
DWI: Diffusion-weighted imaging
EMA: European Medicines Agency
EOAD: Early-onset Alzheimer’s disease
FLAIR: Fluid-attenuated inversion recovery
GFAP: Glial fibrillary acidic protein
GRE: Gradient-recalled echo
IRR: Infusion-related reaction
LOAD: Late-onset Alzheimer’s disease
MCI: Mild cognitive impairment
MMSE: Mini-Mental State Examination
MRI: Magnetic resonance imaging
NfL: Neurofilament light chain
NIA-AA: National Institute on Aging-Alzheimer's Association
PET: Positron emission tomography
PPA: Primary progressive aphasia
RWE: Real-world evidence
SRM: Standardized response mean
SWI: Susceptibility-weighted imaging
